# Exome‐based new allele‐specific PCR markers and transferability for sodicity tolerance in bread wheat ( *Triticum aestivum* L.)

**DOI:** 10.1002/pld3.520

**Published:** 2023-08-18

**Authors:** Roopali Bhoite, Rosemary Smith, Urmil Bansal, Mirza Dowla, Harbans Bariana, Darshan Sharma

**Affiliations:** ^1^ Grains Genetic Improvement Department of Primary Industries and Regional Development South Perth Western Australia Australia; ^2^ The UWA Institute of Agriculture The University of Western Australia Perth Western Australia Australia; ^3^ College of Science, Health, Engineering and Education Murdoch University Perth Western Australia Australia; ^4^ Plant Breeding Institute, School of Life Sciences, Faculty of Science The University of Sydney Cobbitty New South Wales Australia; ^5^ School of Science Western Sydney University Richmond New South Wales Australia

**Keywords:** allele‐specific markers, haplotypes, high‐throughput SNP genotyping, IWGSC RefSeqv1.0, pleiotropic SNP, SNPs, t‐GBS, variant annotation, wheat

## Abstract

Targeted exome‐based genotype by sequencing (t‐GBS), a sequencing technology that tags SNPs and haplotypes in gene‐rich regions was used in previous genome‐wide association studies (GWAS) for sodicity tolerance in bread wheat. Thirty‐nine novel SNPs including 18 haplotypes for yield and yield‐components were identified. The present study aimed at developing SNP‐derived markers by precisely locating new SNPs on ~180 bp allelic sequence of t‐GBS, marker validation, and SNP functional characterization based on its exonic location. We identified unknown locations of significant SNPs/haplotypes by aligning allelic sequences on to IWGSC RefSeqv1.0 on respective chromosomes. Eighteen out of the target 39 SNP locations fulfilled the criteria for producing PCR markers, among which only eight produced polymorphic signals. These eight markers associated with yield, plants m^−2^, heads m^−2^, and harvest index, including a pleiotropic marker for yield, harvest index, and grains/head were validated for its amplification efficiency and phenotypic effects in focused identification germplasm strategy (FIGS) wheat set and a doubled haploid (DH) population (Scepter/IG107116). The phenotypic variation explained by these markers are in the range of 4.1–37.6 in the FIGS population. High throughput PCR‐based genotyping using new markers and association with phenotypes in FIGS wheat set and DH population validated the effect of functional SNP on closely associated genes—calcineurin B‐like‐ and dirigent protein, basic helix–loop–helix (BHLH‐), plant homeodomain (PHD‐) and helix–turn–helix myeloblastosis (HTH myb) type ‐transcription factor. Further, genome‐wide SNP annotation using SnpEff tool confirmed that these SNPs are in gene regulatory regions (upstream, 3′‐UTR, and intron) modifying gene expression and protein‐coding. This integrated approach of marker design for t‐GBS alleles, SNP functional annotation, and high‐throughput genotyping of functional SNP offers translation solutions across crops and complex traits in crop improvement programs.

## 1 INTRODUCTION

Bread wheat is an allohexaploid with a very complex (>80% repetitive DNA) and large genome (approx. 17 Gb, 5× the human genome and 40× the rice genome). The three sub genomes (A, B, and D) have a high level of coding sequence similarity (~95%) between homoeologous genes (He et al., 2019; Huang et al., 2002; Krasileva et al., 2017; Zhou et al., 2020). The high sequence conservation between homoeologous genes coupled with the large genome size makes sequence amplification from a specific genome challenging. However, the suite of recent advances in genomics are now empowering geneticist to better understand tailored gene expressions. The whole genome sequencing technological advances have facilitated a more comprehensive view of diversity and gene function in plants. The availability of chromosome‐based genome sequence of the common wheat (Alaux et al., 2018) and high‐throughput genome sequencing technologies have significantly impacted the wheat genomics research and development landscape (Borrill et al., 2015; Jiao et al., 2011). The development of genotyping platforms such as 90 K iSelect SNP array has fast tracked the discovery and deployment of qualitative and quantitative economic traits in common wheat (Allen et al., 2017; Wang et al., 2014).

Genotyping‐by‐sequencing (GBS) platform enables high‐throughput identification of new variants throughout the genome, contributing to the trait of interest (Deschamps et al., 2012; Ott et al., 2017). Recently, targeted exome‐based genotype by sequencing (t‐GBS) approach was implemented by He et al. (2019) for mapping targeted genes controlling major agronomic traits. The t‐GBS platform generates low rates of missing data due to genome reduction and enables capture of heterozygous and exome‐based alleles, which is crucial for quantitative trait improvements. In a previous investigation, the use of t‐GBS platform has enabled tagging exome‐based de novo SNPs and haplotypes within the diverse wheat lines contributing to yield and yield‐components' tolerance on sodic‐dispersive soils (Sharma et al., 2022). However, the disadvantage of t‐GBS assay, unlike SNP chips, is that it fails to locate the SNPs on 180 bp allelic sequences to enable design of allele‐specific SNP marker, which deters using the t‐GBS functional alleles in practical breeding programs.

Allele‐specific SNP markers offers high‐throughput analysis, low genotyping error rates, detection of co‐dominant inheritance, and high genomic abundance (Allen et al., 2017; Bhoite et al., 2021; Harper et al., 2012). While high throughput genotyping is now becoming an integral component of plant breeding, the conversion of trait‐linked SNPs into allele‐specific markers is challenging particularly in polyploid crop species such as wheat due to the homoeologous and paralogous genomic sequences (Adamski et al., 2020; Ling et al., 2013; Uauy, 2017). Therefore, it is essential to select variants of interest in each homoeolog separately for designing markers specific to the sequence. Advances in genomic resources and functional genomic tools have collectively enabled the understanding of the relationship between homoeologs and identification of homoeolog‐specific variants, which can inform approaches to modulate the response of quantitative traits in polyploid wheat.

Soil sodicity is a complex subsoil constraint affecting all wheat developmental stages and causing loss in productivity (GRDC fact sheet, 2020; Sharma, 2017). Our previous genome‐wide association studies (GWAS) demonstrated soil sodicity as a complex trait and reported number of SNPs and haplotypes contributing to improved yield and yield‐components on sodic soils (Sharma et al., 2022). The objectives of this study were to (1) locate significant de novo SNPs/haplotypes on ~180 bp allelic sequence of t‐GBS platform with respect to IWGSC RefSeqv1.0; (2) design allele‐specific SNP/haplotype markers and check the amplification efficiency of alleles by PCR‐based high throughput genotyping, contributing to sodicity tolerance in wheat (reported by Sharma et al., 2022); (3) annotate and characterize functional variants using SNP effect (SnpEff) pipeline; (4) validate marker effect in full focused identification germplasm strategy (FIGS) wheat set and biparental doubled haploid (DH) population (Scepter/IG107116) by association of high throughput genotype data and phenotype data.

## 2 RESULTS

### 2.1 SNP validation and locating exome‐based SNPs on t‐GBS allelic sequences

**FIGURE 1:**
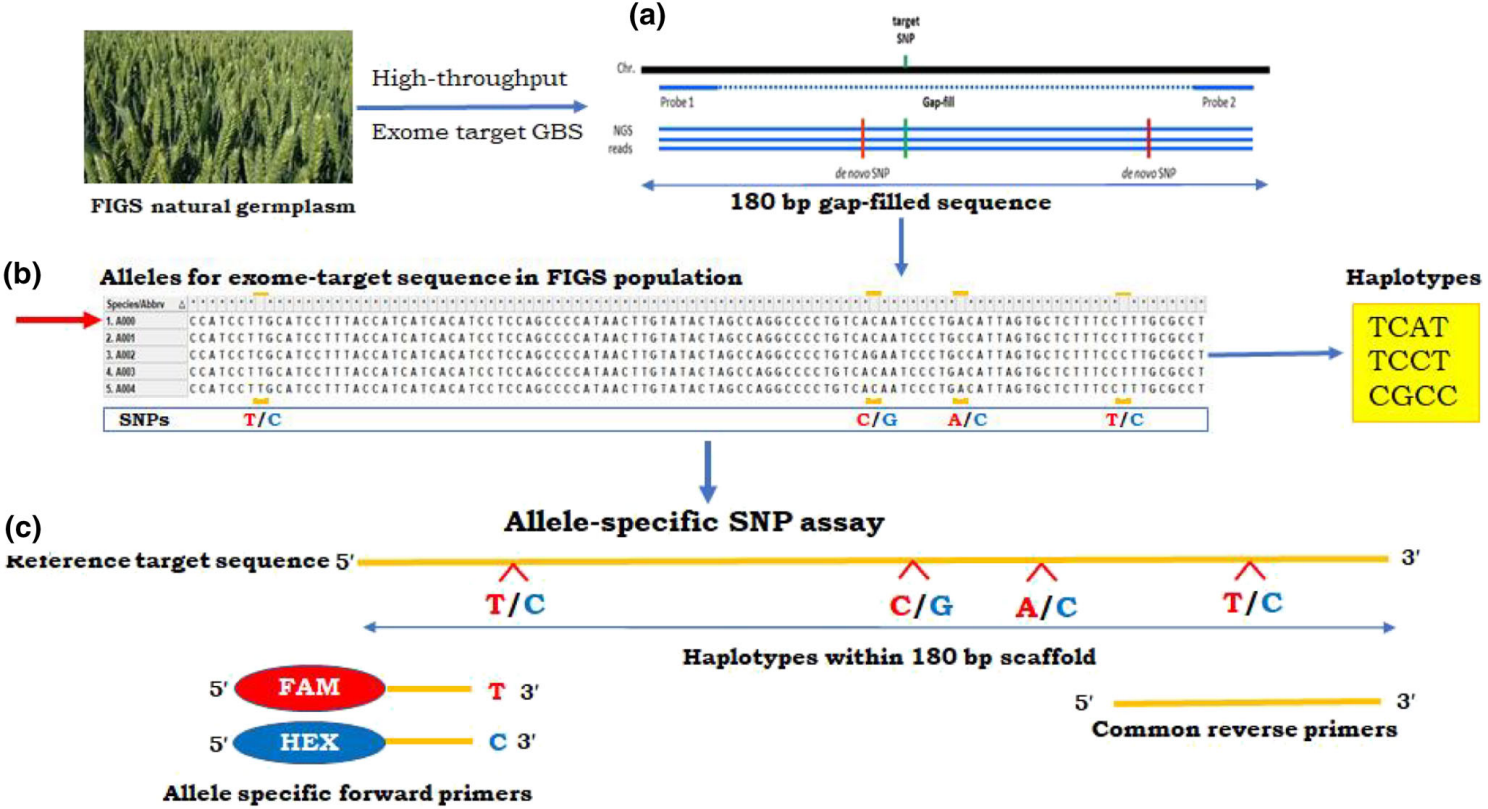
Targeted exome‐based genotype by sequencing (t‐GBS) and allele‐specific marker design. (a) Custom wheat exome t‐GBS assay with target and de novo SNPs within 180 bp sequence gap‐filled allelic sequence between the probes. (b) Genotype calls for SNPs and haplotypes. (c) Design of allele specific markers for de novo SNPs and haplotypes on a stretch of 180 bp gap‐filled exome‐target sequence.

The alignment of respective ~180 bp allelic sequence (Table S1) on to the wheat reference genome (IWGSC RefSeqv1.0) sequence for respective chromosome, resulted in identification of precise location of SNPs and haplotypes (Figure 1, Table S2). Out of 39 significant SNPs, seven SNP locations could not be mapped at the given location on reference sequence and therefore were removed from further analysis. The remaining 32 target variant sequence were used for the design of allele‐specific marker. Among 32, only 18 target variant sequences successfully produced allele specific markers (Table 1).

**TABLE 1:** Significant SNPs with its favorable allele, allele frequency, percentage phenotypic variation, and variant consequence (source: Sharma et al., 2022).

Traits	QTS	Category	SNP	Ref/alt allele	Favorable allele	MAF	PVE (%)	Variant consequence
Yield	YD41–44	Haplotype	scaffold9533;TaGBSv2‐430_551402 + 111	A/G	A	.07	5.4	Synonymous
	YD42		scaffold9533;TaGBSv2‐430_551402 + 94	T/G	T			Missense
	YD43		scaffold9533;TaGBSv2‐430_551402 + 85	G/C	G			Missense
	YD44		scaffold9533;TaGBSv2‐430_551402 + 21	A/G	A			Synonymous
	YD51–52	Haplotype	scaffold47982–2;TaGBSv2‐504_4891482 + 136	T/C	C	.42	4.1	intergenic_region
	YD52		scaffold47982–2;TaGBSv2‐504_4891482 + 110	G/C	C			intergenic_region
	**YD6**	Pleiotropic SNP	scaffold128709;TaGBSv2‐7503_60848 + 9	C/T	C	.06	5.2	intergenic_region
	YD72–73	Haplotype	scaffold4776;TaGBSv2‐8720_295746 + 136	T/C	C			downstream_gene
	YD73		scaffold4776;TaGBSv2‐8720_295746 + 89	G/A	A			downstream_gene
Plants m^−2^	PM2	SNP	scaffold108703;TaGBSv2‐11630_1033239 + 176	A/T	A	.42	14.3	intergenic_region
	PM7	SNP	scaffold10600;TaGBSv2‐11023_3006486 + 120	A/T	T	.47	37.6	intron_variant
Heads m^−2^	HM11–15	Haplotype	scaffold92463;TaGBSv2‐865_5008637 + 37	C/G	T	.45	9.6	3′‐UTR
	HM12		scaffold92463;TaGBSv2‐865_5008637 + 66	T/C	T			3′‐UTR
	HM13		scaffold92463;TaGBSv2‐865_5008637 + 113	G/A	G			3′‐UTR
	HM14		scaffold92463;TaGBSv2‐865_5008637 + 117	A/G	A			3′‐UTR
	HM15		scaffold92463;TaGBSv2‐865_5008637 + 165	T/C	T			3′‐UTR
Harvest index	**HI3/GH1**	Pleiotropic SNP	scaffold128709;TaGBSv2‐7503_60848 + 9	T/C	C	.38	7.2	intergenic_region
	HI4		scaffold158337;TaGBSv2‐7509_168378 + 155	T/C	T	.05	6.7	upstream_gene

*Note*: *YD6/HI3/GH1* are pleiotropic SNPs for yield, harvest index, and grains/head. SNP marker sequence presented in Table S3.

Abbreviations: GH, grains/head; HI, harvest index; HM, heads m^−2^; PM, plants m^−2^; *QTS*, quantitative trait SNP; YD, yield.

### 2.2 Genome‐wide SNP annotation and characterization

**FIGURE 2:**
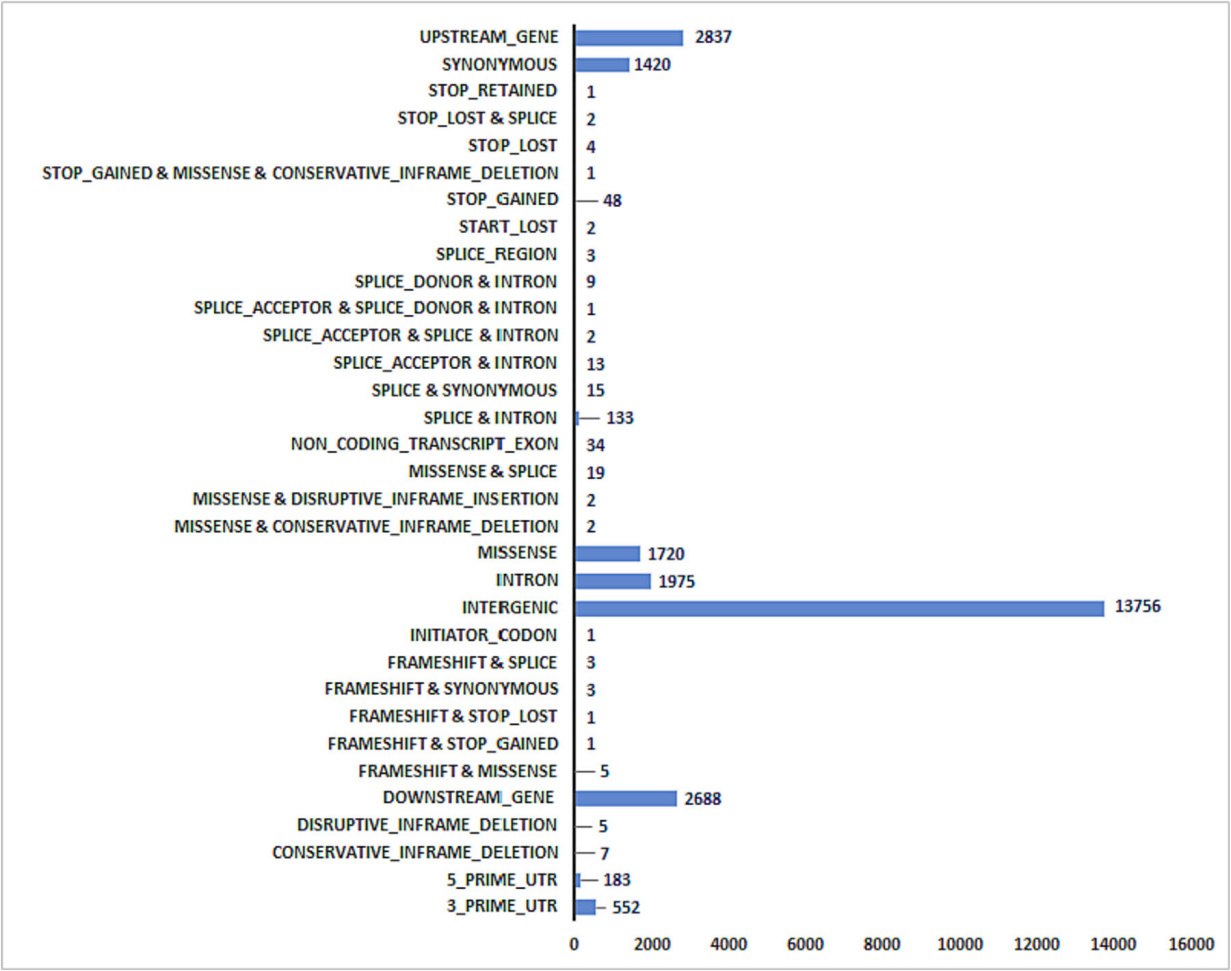
Variant classification of high‐quality 25,448 SNPs identified in 206 focused identification germplasm strategy (FIGS) wheat set. The number of SNPs in each class is shown beside the horizontal bar.

The final variant calling output file (VCF) file after quality filtering resulted in 25,448 variants. The variants were annotated into 33 different classes based on their predicted effects on protein function (Figure 2). A complete list of SnpEff output for 25,448 variants are presented in Table S3. The largest number of SNPs are in intergenic regions (54%), followed by upstream (11.1%) and downstream genes (10.5%), and intronic regions (7.7%). Synonymous and missense accounted for 5.6% and 6.7%, respectively, followed by 3′ UTR (2.1%) and 5′ UTR (.7%). The significant SNPs/haplotypes contributing to yield and yield‐components on sodic‐dispersive soils (Sharma et al., 2022) were further selected for detailed investigation. Most of the SNPs associated with sodic‐dispersive tolerance are identified in regulatory regions (upstream, 3′‐UTR and intron) with few missense mutations (Tables 1 and 5). The successfully amplified markers for yield, plants m^−2^, heads m^−2^, and harvest index are presented in Table 5, with its impact type, variant consequence, associated genes, and transcription factors (TFs).

### 2.3 Allele‐specific PCR markers and polymorphic clusters

**FIGURE 3:**
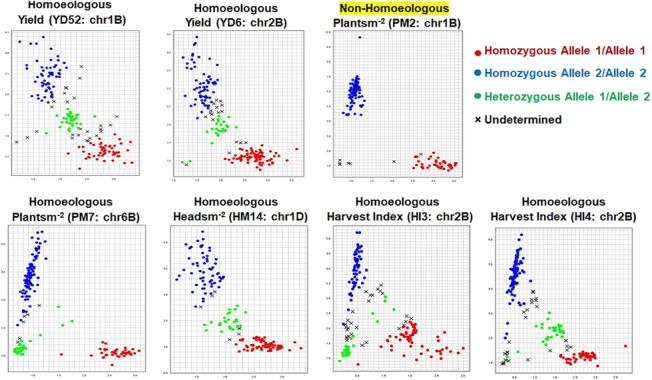
Allele discrimination plots of SNP markers for full focused identification germplasm strategy (FIGS) wheat set (192 lines) for enhancement of yield (YD), plants m^−2^ (PM), heads m^−2^ (HM), harvest index (HI). The HEX and FAM florescent signals are represented as homozygous allele 1 (red) and allele 2 (blue), respectively, and heterozygous allele (green). *YD6* is a pleiotropic SNP contributing to grains/head (*GH1*) and harvest index (*HI3*); *PM2* is a part of haplotype at 53737652–53737716 on chromosome 1B; HM14 is a part of haplotype at 356142374 on chromosome 1D; *HI3* is a part of haplotype at 34083794 on chromosome 2B. The positions of SNPs are presented in Table 2. The clusters representing alleles as follows: *YD52* (red, CC [favorable]; green, GC; blue, GG), *YD6* (red, CC [favorable]; green, CT; blue, TT), *PM2* (red, TT; blue, AA [favorable]), *PM7* (red, TT [favorable]; blue, AA), HM14 (red, GG; green, GA; blue, AA [favorable]), *HM15* (green, CT; blue, TT [favorable]), *HI3* (red, CC [favorable]; green, CT; blue, TT), *HI4* (red, CC; blue, TT [favorable]). *YD6/HI3/GH1* are pleiotropic SNPs for yield, harvest index, and grains/head. GH1 plot is not presented because of high phenotype missing value.

FIGS wheat set (192) was genotyped with 18 allele‐specific markers (Table S4). The allele discrimination plots for full FIGS set are presented in Figure 3. Markers for yield—*YD52* (1B: 411775364), *YD6* (2B: 34083794); plants m^−2^—*PM7* (6B: 486517837), *PM2* (1B: 53737652); heads m^−2^—*HM14* (1D: 356142374); harvest index—*HI3* (2B: 34083794), *HI4* (1D: 41415831) produced allele‐specific polymorphic clusters. Scepter and IG107116, the parents of DH population, produced polymorphic allelic signals for yield (*YD52*, *YD6*), plants m^−2^ (*PM7*, *PM2*) (Figure S1). The successfully amplified primer sequence with its SNP type (homoeologous and non‐homoeologous), chromosome location, position, and orientation are presented in Table 2. The *YD6/HI3/GH1* allele identified on chromosome 2B is pleiotropic for yield, harvest index, and grains/head, respectively; therefore, they have common allele‐specific SNP marker. All the SNP markers, except for *PM2* were homoeologous (multiple gene copies in A, B, and D genome) having both homozygous and heterozygous calls. The marker for PM2 on chromosome 1B was non‐homoeologous (single copy) having two clear homozygous clusters.

**TABLE 2:** Primer sequences of successfully amplified allele‐specific SNP markers and physical location of SNPs based on IWGSC RefSeq v1.0.

Marker	chr	SNP_type	Physical position	Forward 1 (Tm°C)	Forward 2 (Tm°C)	Common reverse (Tm°C)	Orientation
YD52	1B	Homoeologous	411,775,364	cgtttgtggttgttttgcaagtaG (59.9)	cgtttgtggttgttttgcaagtaC (60.2)	gtcgctggttggtccaatct (60.3)	Reverse
YD6/HI3/GH1	2B	Homoeologous	34,083,794	gggcaggatgaatgaccttaaA (58.3)	gggcaggatgaatgaccttaaG (58.7)	Gccaagccttagtttagtttgca (59.9)	Reverse
PM2	1B	Non‐homoeologous	53,737,652	tcctgtaactgttgcaactacgA (59.9)	tcctgtaactgttgcaactacgT (60.1)	ataacttgagtttagagaagctcct (57.5)	Reverse
PM7	6B	Homoeologous	486,517,837	accagcagagttctgaaaaataaaA (57.6)	accagcagagttctgaaaaataaaT (57.4)	cctggaacttgtacatttcagc (57.5)	Reverse
HM14	1D	Homoeologous	356,142,374	ggtcctttcagttgaagaacgT (58.7)	ggtcctttcagttgaagaacgC (60)	tcatgttggtccgactcttc (57.2)	Reverse
HI4	2B	Homoeologous	41,415,831	tgacagtgacgcatatcaaagtT (58.6)	tgacagtgacgcatatcaaagtC (59)	cgcagggccttatctaatgc (58.8)	Forward

*Note*: *YD6/HI3/GH1* are pleiotropic SNPs for yield, Harvest index and Grains/head.

Abbreviations: chr, chromosome; GH, grains/head; HI, harvest index; HM, heads m^−2^; PM, plants m^−2^; Tm, melting temperature; YD, yield.

### 2.4 High throughput genotyping of functional SNPs

Genotyping full FIGS set and DH population using new SNP markers resulted in homozygous and heterozygous allelic signals (Figure 3), which were grouped separately and correlated with phenotypic values (Table 3). The least square difference (LSD) estimates calculated based on phenotypic values for favorable allele group for yield *(YD52*, *YD6)*, plants m^−2^ *(PM7)*, heads m^−2^ *(HM14)*, harvest index *(HI3*, *HI4)* except, for plants m^−2^ (*PM2)* were less than the absolute phenotypic difference between alternative allele groups, indicating the significance of favorable allele contributing to yield and yield‐components. The average phenotypic values of favorable allele and alternative allele groups in DH lines for *PM2*, *PM7*, *YD6*, and *YD52* are presented in Table 4. The DH lines differed in average grain weight production between allelic groups at *p* < .05. Overall, the presence of favorable alleles increased grain production.

**TABLE 3:** Least significant difference (LSD) estimates for homozygous (allele 1 and 2) and heterozygous allele (allele 3) groups for SNP markers and phenotypic validation in FIGS population.

Trait	QTS	PVE (%)	Ref/alt allele	FIGS wheat panel			
				Phenotypic mean ICT	Absolute of average	LSD	Means differ at *p* ≤ .05
Yield (YD)	YD52	4.1	**CC (favorable)**	.010	.091 ǀG‐Sǀ	.085 (abs of average > LSD)	**Allele C > G**
			GG	−.002	.012 ǀG‐Cǀ	.039 (abs of average < LSD)	Not significant
			GC (S)	−.084	.095 ǀC‐Sǀ	.086 (abs of average > LSD)	Allele C > S
	YD6	5.2	**C (favorable)**	.062	.131 ǀC‐Tǀ	.05 (abs of average > LSD)	**Allele C > T**
			TT	−.069	.074 ǀC‐Yǀ	.039 (abs of average > LSD)	Allele C > Y
			CT (Y)	−.012	.056 ǀT‐Yǀ	.050 (abs of average > LSD)	Allele Y > T
Plants m^−2^ (PM)	PM2	14.3	**AA (favorable)**	.256	.463 ǀA‐Tǀ	.619 (abs of average < LSD)	**Not significant**
			TT	−.207			
	PM7	37.6	**TT (favorable)**	1.20	1.55 ǀA‐Tǀ	.872 (abs of average > LSD)	**Allele T > A**
			AA	−.35			
Heads m^−2^ (HM)	HM14	9.6	**AA (favorable)**	7.81	65.79 ǀA‐Gǀ	31.60 (abs of average > LSD)	**Allele A > G**
			GG	−57.97	7.83 ǀA‐Rǀ	11.08 (abs of average < LSD)	Not significant
			AG (R)	−.017	57.95 ǀG‐Rǀ	29.99 (abs of average > LSD)	Allele R > G
Harvest index (HI)	HI3	7.2	**CC (favorable)**	.011	.014 ǀC‐Tǀ	.009 (abs of average > LSD)	Allele C > T
			TT	−.003	.011 ǀC‐Yǀ	.007 (abs of average > LSD)	**Allele C > Y**
			CT (Y)	.0001	.003 ǀT‐Yǀ	.009 (abs of average < LSD)	Not significant
	HI4	6.7	**TT (favorable)**	.005	.025 ǀT‐Cǀ	.016 (abs of average > LSD)	**Allele T > C**
			CC	−.019	.016 ǀT‐Yǀ	.03 (abs of average < LSD)	Not significant
			TC (Y)	−.010	.008 ǀC‐Yǀ	.034 (abs of average < LSD)	Not significant

*Note*: *YD6/HI3/GH1* are pleiotropic SNPs for yield, harvest index, and grains head^−1^.

Abbreviations: FIGS, focused identification germplasm strategy; PVE, phenotypic variance explained; QTS, quantitative trait SNPs.

**TABLE 4:** Phenotypic validation of polymorphic markers in scepter/IG107116 doubled haploid population.

QTSs	DH population	Ref/alt allele	Grain Wt_g	*p*‐Value^a^	Effect (%)^b^
YD6	Scepter/IG107116	CC (favorable)	85.26	4.56E‐05*	53.2137
		CT	39.89		
YD52	Scepter/IG107116	CC (favorable)	80.25	.026*	29.86916
		CG	56.28		
PM2	Scepter/IG107116	AA (favorable)	64.74148	.047*	5.133039
		TT	61.41828		
PM7	Scepter/IG107116	TT (favorable)	99.71556	.004 (T vs. A)*	56.72691
		AA	43.15	.083 (A vs. TA)	−40.3086
		TA	72.28848	.064 (TA vs. T)	−27.5053

Abbreviations: QTS, quantitative trait SNPs; *YD6/HI3/GH1* are pleiotropic SNPs for yield, harvest index, and grains head^−1^.

^a^ Student's *t*‐test (*p* < .05) was used to identify differences between allele groups. Favorable allele validated from fluorescent signals through SNP genotyping assays.

^b^ Relative yield increase with respect to alternative allele group.

* Significance at *p* < .05.

**FIGURE 4:**
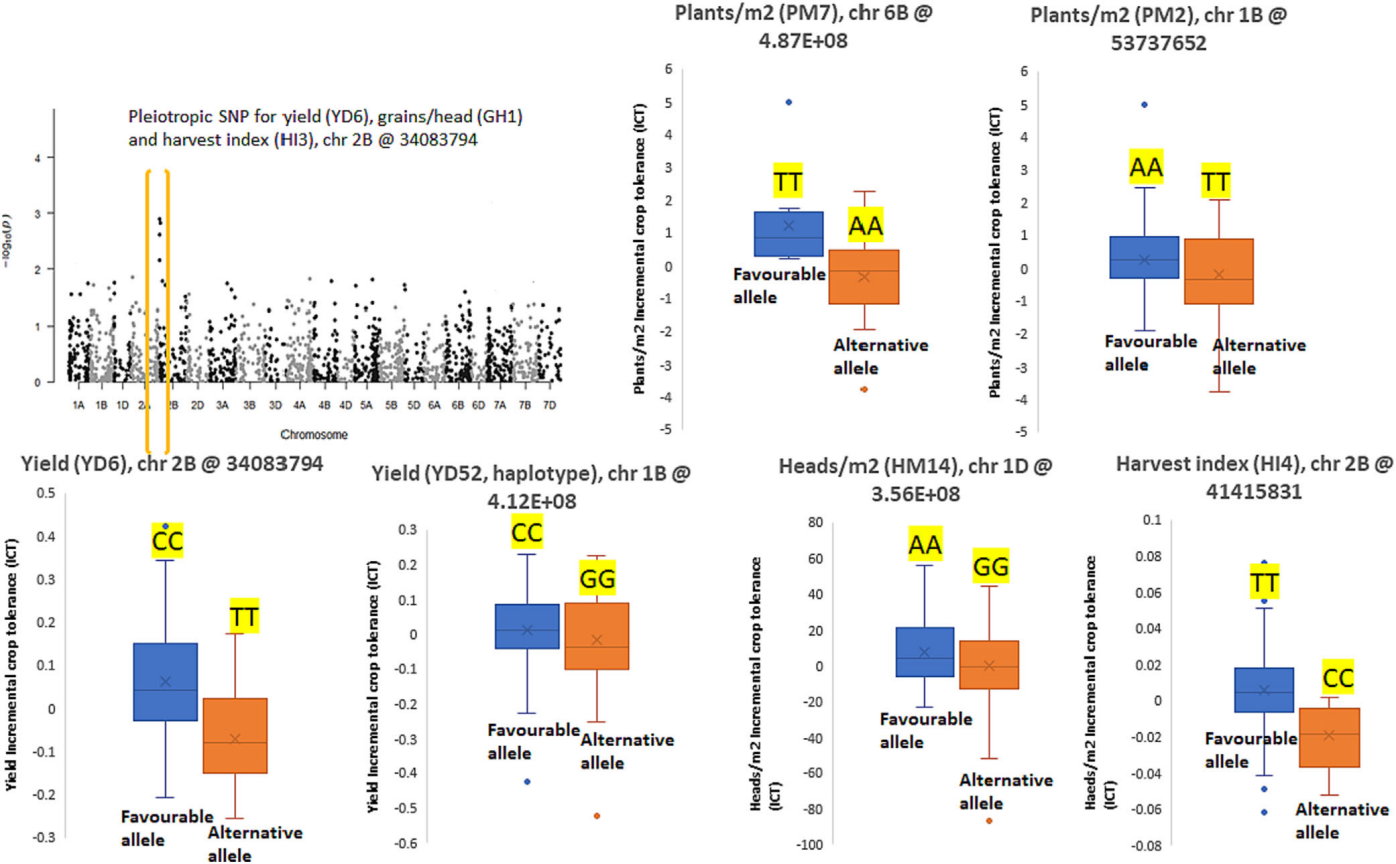
Box plots representing phenotypic variation of SNP markers in focused identification germplasm strategy wheat set. First box represents favorable homozygous allele, followed by homozygous alternative allele for yield (*YD6*: chr 2B [*YD6/HI3/GH1* are pleiotropic SNPs for yield, harvest index, and grains/head]; *YD52*: chr 1B), Plants m^−2^ (*PM2*: chr 1B, *PM7*: chr 6B), Heads m^−2^ (*HM14*: chr 1D, *HM15*: chr 1D), and harvest index (*HI3*: chr 2B, *HI4*: chr 2B). Manhattan plot is sourced from previous genome‐wide association studies (GWAS) publication (Sharma et al., 2022).

The genes (calcineurin B‐like‐ and dirigent protein, basic helix–loop–helix [BHLH‐], plant homeodomain [PHD‐] and helix–turn–helix myeloblastosis [HTH myb] type TF) closely associated with functional SNPs are presented in Table 5. The box plots in Figure 4 represents phenotypic variations for all successfully amplified markers at a given SNP location associated with the genes stated above. The favorable homozygous allelic effect was presented in the first box followed by unfavorable allelic effect in the second box to promote uniform visualizations. The pleiotropic marker for yield (*YD6*) and harvest index (*HI3*), and haplotype for grains/head (*GH1*) on chromosome 2B: 34083794, is 14.8 kb away from the gene encoding dirigent proteins and lines having favorable allele CC had improved yield and yield‐components compared to alternative allele TT. Marker for yield (*YD52*) on chromosome 1B: 411775364, is 45.8 kb away from the gene encoding calcineurin B‐like protein and the FIGS lines having favorable allele CC had improved yield compared to lines having alternative allele GG. The marker for plants m^−2^ (*PM7*) is an overlapping intron gene regulatory variant, encoding BHLH domain TF and the lines having favorable allele TT improved wheat establishment compared to alternative allele AA. The marker for plants m^−2^ (*PM2*) is associated with uncharacterized protein and favorable allele AA on chromosome 1B: 53737652 seemed beneficial to allele TT for greater wheat establishment on sodic soils. The marker for heads m^−2^ (*HM14*) is an overlapping 3′‐UTR gene regulatory variant, encoding PHD‐type domain TF and the lines having favorable allele AA improved heads m^−2^ compared to alternative allele GG. The marker for harvest index (*HI4*) is an upsteam gene regulatory variant, encoding HTH myb‐type TF and the lines having favorable allele TT improved harvest index compared to alternative allele CC. In silico gene expression analysis, it is revealed that most of the genes have the highest expression at critical wheat growth stages (germination, stem elongation, anthesis, and grain development) (Table 5).

**TABLE 5:** Successfully amplified allele‐specific SNPs and haplotypes with its impact type on genes and variant consequence.

QTS	Traes gene ID	Impact type	Variant consequence	BioType	Candidate genes and transcription factors
YD52 (haplotype)	TraesCS1B02G229200‐TraesCS1B02G229300	Modifier	Intergenic region	‐	Calcineurin B‐like protein 1
YD6, GH1, HI3 (pleiotropic SNP and haplotype)	TraesCS2B02G067400‐TraesCS2B02G067500	Modifier	Intergenic region	‐	Dirigent protein—stem elongation
PM2 (SNP)	TraesCS1B02G069000‐snoR64	Modifier	Intergenic region	Protein coding	Uncharacterized protein
PM7 (SNP)	TraesCS6B02G270200	Modifier	Intron variant	Protein coding	BHLH domain‐containing protein
HM14 and HM15 (haplotype)	TraesCS1D02G262200	Modifier	3′‐UTR	Protein coding	PHD‐type domain protein
HI4	TraesCS2B02G076500	Modifier	Upstream gene	Protein coding	HTH myb‐type uncharacterized protein

*Note*: YD6/HI3/GH1 are pleiotropic SNPs for yield, harvest index, and grains head^−1^.

Abbreviations: GH, grains head^−1^; HI, harvest index; HM, heads m^−2^; PM, plants m^−2^; QTS, quantitative trait SNPs; YD, yield.

## 3 DISCUSSIONS

### 3.1 High LD exome‐based alleles and marker design

The integration of exome‐based wheat genome sequences and genome‐wide variant annotation and characterization has enabled tagging new functional variants (SNPs/haplotypes) in gene‐rich regions contributing to yield and yield‐components. The high linkage disequilibrium (LD) novel haplotypes (spanning ~180 bp scaffolds) infer maximum genotypic variation for a given gene, without any computation estimation. The local patterns of LD and variant loci in gene‐rich regions are rich genetic resources to improve gene expression contributing to yield and yield‐components on sodic soils. Haplotype‐based breeding has a scope of incorporating desirable alleles of candidate genes either by enhancement of gene expression rate or change in desired protein characteristics contributing to complex trait expression (Bhat et al., 2021; Stram, 2017). In general, gene‐associated SNPs and high LD haplotype alleles identified within ~180 bp gene‐rich (exome) regions represented maximum genotypic variation, which will also rapidly improve genetic gain in breeding complex traits.

The allele‐specific PCR markers developed for all developmental stages are very efficient and quick ways to ascertain the presence of desirable alleles in plant breeding for complex traits (Sun et al., 2010). They have an edge over the conventional phenotyping as these are neither affected by growth stages of plants nor by environmental changes. Although, t‐GBS platform (He et al., 2019) identified new variants contributing to yield and yield‐components on sodic‐dispersive soils, the platform does not infer the exact location of new variants on ~180 bp allelic sequences (Table S1). Therefore, in the present investigation, we identified the allelic location by aligning t‐GBS allelic sequence on to the reference chromosomal sequence (IWGSC RefSeq v1.0; (Alaux et al., 2018; Table S2). The SNP markers were then designed around the allelic location contributing to yield and yield‐components tolerance on sodic soils. Some of the SNP location did not support marker design due to failed optimal GC content, high hair‐pin loop stability, marker in a repetitive sequence, unacceptable product size, nonspecific forward chromosome, and many homoeologs (Grewal et al., 2020; Ramirez‐Gonzalez et al., 2015).

### 3.2 Homoeologous and non‐homoeologous SNP markers in polyploid wheat

Advances in genomic resources and functional genomic tools have collectively enabled the understanding of relationship between homoeologues and identification of homoeolog‐specific variants that can inform approaches to modulate the response of quantitative traits in polyploid wheat. The present investigation identified both non‐homoeologous and homoeologous markers. In a complex wheat genome, homoeologous markers produce both homozygous and heterozygous calls, because hexaploidy wheat (*T.aestivum*, 2*n =* 6*×* = 42, genome constitution AABBDD) is an allopolyploid that was formed through hybridization and successive chromosome doubling of three ancestral diploid species (2*n* = 14), *T.urartu* (AA), *Aegilops speltoides* (BB), and *Ae. squarrosa* (DD) (Huang et al., 2002). Chromosome mapping using aneuploid lines of hexaploidy wheat infer that the genes coexist on homoeologous chromosome groups (Nomura et al., 2003), resulting in both homozygous and heterozygous calls (Figure 3). For homoeologous markers, there is always a chance of presence of alternative alleles associated with homoeologous genes for a respective wheat line, which results in amplification of both the alleles, producing homozygous and heterozygous calls. However, the present study also reported a non‐homoeologous marker for plants m^−2^ (*PM2*), with a single gene copy on the chromosome 1B, and clear homozygous clusters could be visualized (Figure 3).

### 3.3 Functional genomics and SNP characterization

Functional SNPs for significant wheat developmental stages are among major tools to ascertain the presence of desirable alleles in plant breeding to enhance complex traits (Cingolani et al., 2012; Sarkar & Maranas, 2020) like sodicity tolerance. The variant annotation and functional characterization have identified the location of variants (SNPs/haplotypes) in exome and impact on protein coding (Table 5). SNPs identified in gene regulatory and intronic regions (BHLH‐, PHD‐, and HTH myb type TF) in this study for Plants m^−2^, Heads m^−2^, and harvest index encode TFs, known to improve abiotic stress tolerance without a yield penalty (Mickelbart et al., 2015). SNPs identified in regulatory regions have significant roles in the activation of effector molecules directly involved in mitigating stress (Cingolani et al., 2012; Cummings et al., 2019; Vij & Tyagi, 2007; Watanabe et al., 2019; Yamaguchi‐Shinozaki & Shinozaki, 2006; Yang & Wang, 2015; Yao et al., 2014). These functional SNPs could be used as targets for molecular breeding and high‐throughput identification of target mutations in wheat improvement programs. SNPs having missense mutations and in regulatory regions (3′‐UTR, upsteam and downstream; Table 1) could be further investigated for designing targets for gene editing to enhance complex traits.

### 3.4 High‐throughput genotyping using new exome‐based PCR markers

The high through‐put genotyping using functional SNP markers (closely associated with genes) in full FIGS set and correlations with phenotypic values (Figure 4, Table 3) validated genes encoding calcineurin B‐like (Borjigin et al., 2021; Gao et al., 2015) and dirigent‐protein (Khan et al., 2018; Li et al., 2017) and TFs—BHLH, PHD‐type, HTH myb (Table 5) (Bhoite et al., 2021; Mickelbart et al., 2015; Roy, 2016). The exome‐based alleles/SNPs are present in gene‐rich regions, regulating the function of candidate genes; therefore, the positive effect of candidate genes associated with favorable allele/SNP for trait expression was also confirmed. It is well known that functional gene validation through molecular techniques such as gene cloning and knocking out of genes are required to confirm the effect of genes on trait expression. However, in the case of complex quantitative trait like soil sodicity, recording phenotypic effect of multiple minor‐modifier genes throughout the wheat developmental stages has been a challenging task with no expression of distinct phenotypes. Also, it is difficult to imitate complex subsoil constraints in pot studies. Using exome‐based functional SNPs is paramount to investigate genetics of complex quantitative traits. Therefore, in our previous GWAS on sodicity tolerance (Sharma et al., 2022), we used exome‐based alleles, which is directly/indirectly involved in gene expression and protein coding.

Sodic‐dispersive soils have high levels of sodium ions (Na^+^) and clay. The negatively charged clay particles attract positively charged Na^+^ ions, forming a massive duplex and dispersive soil, with higher pH (>8) (Arif et al., 2020; Orton et al., 2018). As a result, wheat development and root patterning are disturbed, and acclimation response depends on redox and reactive oxygen species signaling, calcium, and plant hormones. Calcineurin B‐like protein is a Ca^2+^ ion sensor that increase intracellular Ca^+2^ ion concentration, channel K^+^ ions, and sequester excess Na^+^ ions into vacuolar space (Borjigin et al., 2021; Gao et al., 2015). This corroborative effort improves K^+^/Na^+^ equilibrium and cellular ionic homeostasis. The dirigent proteins predominantly express in stems and have roles in biosynthesis of lignin‐like molecules, secondary metabolism, and fiber synthesis. This metabolic plasticity is a defense response against abiotic stresses (Khan et al., 2018; Li et al., 2017). The TFs (BHLH domain, PHD‐, and HTH myb‐type) play a critical role in plant growth and development and are significantly involved in several abiotic stress tolerance mechanisms and reactive oxygen species signaling. These TFs are usually co‐expressed to enhance grain size and yield under abiotic stress in cereal crops (Bhoite et al., 2021; Roy, 2016). The reported genes are highly expressed during germination and anthesis, the major target constraint stage for sodicity (Sharma et al., 2022). Therefore, the reported genes are best candidates for wheat improvement on sodic soils.

Quantitative traits are governed by many minor genes and deciphering gene function based on gene‐by‐gene functional validation is arduous and impractical (Mackay, 2001; MacKay et al., 2009). The rapid gene validation approach presented here is highly applicable to determine the effects of multiple minor gene effect having cumulative effects on complex trait expressions. The present investigation of tagging local patterns of LD (haplotypes) and variant loci on t‐GBS allelic sequences enabled capture of maximum genotypic variations with high heritability for improving yield and yield‐components tolerance. These rich genetic resources could be effectively used in quantitative wheat improvements. The diagnostic markers can be used in PCR‐based high throughput genotyping instead of gel‐based genotyping to rapidly detect favorable genomic variation (SNPs) among breeding materials, introgressing/pyramiding alleles in high‐yielding backgrounds, and high‐throughput identification of target functional mutations in crop breeding. The exome‐based SNP validation, high‐throughput marker development, and gene validation strategy used in this study can be broadly applied to improve any qualitative and/or quantitative traits across crops.

## 4 MATERIALS AND METHODS

### 4.1 Plant material and DNA extraction

One hundred ninety‐two wheat accessions from a natural diverse FIGS population and 88 lines from a DH population (Scepter/IG107116) were used in the present study. FIGS are sorted lines from 24 countries for tolerance to subsoil challenges including high salinity and ‐pH (FIGS‐ICARDA, 2021; McDonald & Schilling, 2019).

Our previous GWAS on sodicity tolerance identified significant SNPs/haplotypes (Sharma et al., 2022), and the present study focused on design of allele‐specific SNP markers and validation of SNP effect in the entire FIGS wheat set using high‐throughput PCR‐based genotyping assay. Genomic DNA was extracted using a modified Sodium Dodecyl Sulfate (SDS) method and quantified using a Nanodrop ND‐1000 spectrophotometer (Thermo Scientific, USA). Further, markers were tested for polymorphism on DH population parents (Scepter and IG107116) and DH lines were screened for marker effect. The parents for DH population were selected based on the observed variance for soil sodicity tolerance under field conditions. Scepter is a predominant variety in Western Australia and performs well on sodic soils (Dowla et al., 2021) while IG107116 is a landrace from Iran. The DH population was phenotyped in Merredin on sodic site during the winter season in 2021 in 50 cm row plots.

### 4.2 Locating new exome‐based SNPs with respect to IWGSC RefSeqv1.0

The t‐GBS genotyping captured significant new SNPs/haplotypes (MAF ≥ .05) at different unknown locations on ~180 bp allelic sequences for yield and yield‐components (Table S1). The possible mutations in allelic sequences that improve the trait value were considered as potential high LD haplotypes (Bhat et al., 2021; Stram, 2017) (Figure 1C). To design a marker, the precise location of SNPs/haplotypes should be known with respect to the reference sequence. Therefore, each allelic sequence identified for a trait was aligned on to IWGSC RefSeqv1.0 on respective chromosome as presented in Table S2. IWGSC RefSeqv1.0 sequence for respective chromosome (Figure 1) were retrieved from URGI (https://wheat-urgi.versailles.inra.fr/Seq-Repository) for Chinese Spring v1.0 (Alaux et al., 2018). The parameters, alignment coverage, and identities greater than 99% were used for defining a valid blast hit on chromosomes specific to GWAS signal.

### 4.3 Design of exome‐specific PCR markers

Chromosom‐specific primers were designed using the PolyMarker bioinformatics pipeline (http://www.polymarker.info/; Ramirez‐Gonzalez et al., 2015), presented in the Tables 2 and S3. For any given SNP location on allelic sequence, a minimum of 60 bp flanking reference sequence (IWGSC RefSeqv1.0, Alaux et al., 2018) was used to design allele‐specific forward primers. Two different tail sequences, GAAGGTGACCAAGTTCATGCT and GAAGGTCGGAGTCAACGGATT, were added to the 5′‐end of each forward primer to match with the FAM‐ and HEX‐fluorescence‐dye‐labeled sequences, respectively, in the allele‐specific reaction mix. Homozygous calls were represented by FAM‐red and HEX‐blue signals and heterozygous calls were represented by green signals.

### 4.4 High throughput SNP genotyping

FIGS wheat and DH population lines were genotyped using PCR allele competitive extension (PACE) assay (Danilova et al., 2019). PCR was conducted following conditions: hot start at 94°C for 15 min, 10 touchdown cycles (94°C for 20 s; touchdown at 61°C, 0.6°C per cycle, 20 s), 35 cycles of amplification (94°C for 20 s; 55°C for 60 s), and the read stage at 30°C for 60 s. PCR products were scanned in a Applied Biosystems® (ViiA 7). Allele discrimination and florescent signals of SNP markers were assessed in the QuantStudio 5 software. The allele calls with quality score >95 was considered for high‐throughput gene validation. The FIGS lines were genotyped using new gene‐associated functional markers, and favorable and alternative alleles were grouped separately. The genotypes and phenotypes were correlated to elucidate the effect of gene‐specific favorable allele on phenotypic expression. The polymorphic markers between Scepter and IG107116 were tested for marker effect in DH lines. Lines were grouped based on allelic calls (favorable and alternative alleles) and Student's *t*‐test was applied to test the significance of phenotypic differences between the groups.

### 4.5 Exome‐based SNP annotation and function

The VCF from the previous GWAS study was filtered to extract high‐quality variants following parameters: minor allele frequency >.05% and genotype information >80%. The final VCF file was subjected to variant characterization using the SnpEff (version 4.3K; Cingolani et al., 2012). The characterized variants were further studied to analyze its effect on protein coding using the wheat genome binary database configured in SnpEff tools. The effect of variants on protein function were classified as high (frame shifts, addition/deletion of stop codons, etc.), moderate (codon change/deletion/insertion, etc.), low (synonymous changes, etc.), and modifier (changes outside coding regions, etc.), providing a simple assessment of the putative impact of the variant on gene expression and protein coding (Cummings et al., 2019; Vij & Tyagi, 2007; Yang & Wang, 2015).

## AUTHOR CONTRIBUTIONS

Darshan Sharma and Roopali Bhoite conceived the idea and study design. Roopali Bhoite carried out experiments, and genomic and statistical data analysis. Rosemary Smith and Mirza Dowla oversaw the seed material collection. Urmil Bansal and Harbans Bariana offered crucial details about genotyping and marker development. Roopali Bhoite drafted the article, with critical revisions from Darshan Sharma, Harbans Bariana, and Urmil Bansal. All authors reviewed and approved the final manuscript.

## CONFLICT OF INTEREST STATEMENT

The Authors did not report any conflict of interest.

## PEER REVIEW

The peer review history for this article is available in the supporting information for this article.

## Supporting information


**Figure S1.** Parental polymorphisms (Scepter/IG107116 and Westonia Nax5991/IG107116) for allele‐specific markers, for plantsm^−2^ and yield. *YD6/HI3/GH1* are pleiotropic SNPs for yield, Harvest index and Grains/head.

**Table S1.** Significant Quantitative trait SNPs and haplotypes identified for yield and yield‐components (Sharma et al., 2022), with their allelic sequences.
**Table S2.** Mapping of favourable allele (SNPs and haplotypes) location with respect to reference sequence (IWGSC RefSeqv1.0) for marker design.
**Table S3.** Characterization of 25,471 genome‐wide SNPs identified in 206 FIGS population using SnpEff tool.
**Table S4.** Successfully designed allele‐specific primers for SNPs and haplotypes for sodic‐dispersive soil tolerance.

**Data S1.** Peer Review.

## Data Availability

Genotype by sequencing data and significant allelic sequences for yield and yield‐components are presented in supplementary tables.
