# Supplementary material for: Exome‐based new allele‐specific PCR markers and transferability for sodicity tolerance in bread wheat ( Triticum aestivum L.)
Source: Plant Direct. 2023 Aug 18;7(8):e520. doi: 10.1002/pld3.520 (PMC10435944; doi:10.1002/pld3.520)
Supplement: Supplementary file 1 — Figure S1. Parental polymorphisms (Scepter/IG107116 and Westonia Nax5991/IG107116) for allele‐specific markers, for plantsm−2 and yield. YD6/HI3/GH1 are pleiotropic SNPs for yield, Harvest index and Grains/head. [file PLD3-7-e520-s001.pdf]

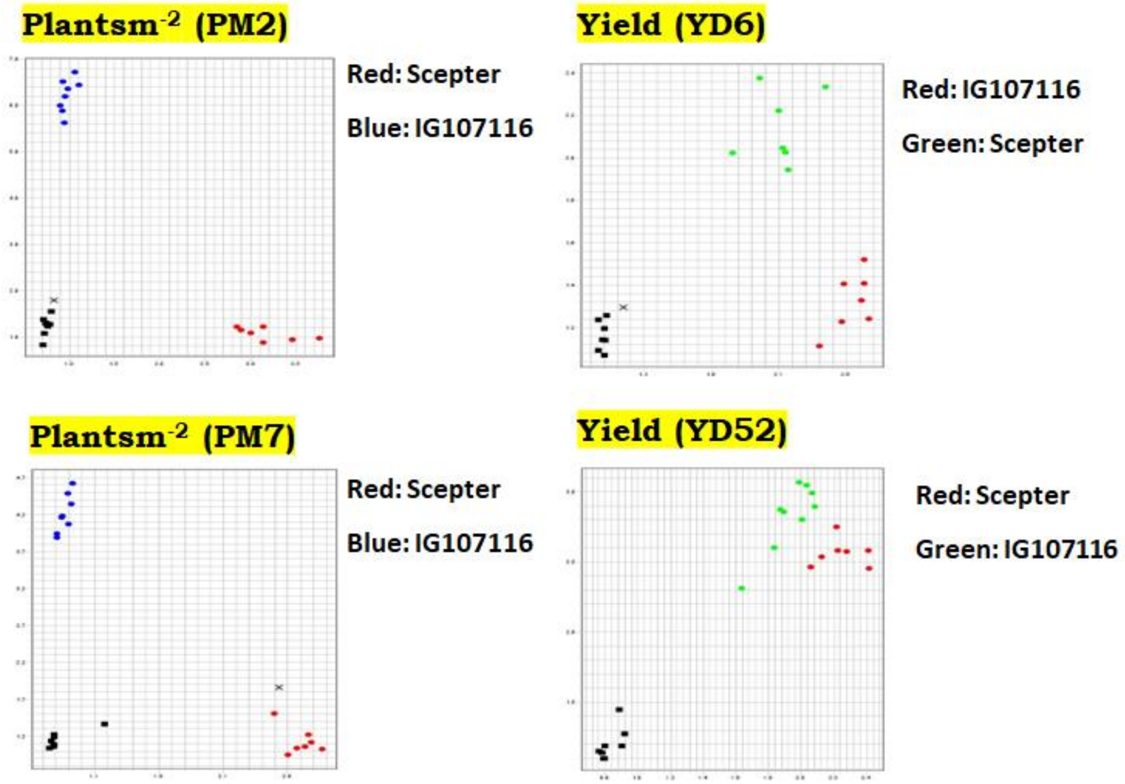

Fig. S2 Parental polymorphisms (Scepter/IG107116 and Westonia Nax5991/IG107116) for allele-specific markers, for plantsm^{-2} and yield. *YD6/HI3/GH1* are pleiotropic SNPs for yield, Harvest index and Grainshead^{-1}
