# Supplementary material for: Exome‐based new allele‐specific PCR markers and transferability for sodicity tolerance in bread wheat ( Triticum aestivum L.)
Source: Plant Direct. 2023 Aug 18;7(8):e520. doi: 10.1002/pld3.520 (PMC10435944; doi:10.1002/pld3.520)
Supplement: Supplementary file 3 — Data S1. Peer Review. [file PLD3-7-e520-s002.docx]

**DECISION LETTER – Round 1**

June 29, 2023

Darshan Sharma
Western Australia Department of Primary Industries and Regional Development Agriculture and Food
3 Baron Hay Court
Bentley Delivery Centre 6151
Australia


RE: Exome-based novel SNPs/haplotypes, allele-specific markers and transferability for sodicity tolerance in wheat

Dear Dr. Sharma:

Thank you for submitting to Plant Direct and using our transfer feature. All required reviews have been returned and we have now finished our evaluation of your manuscript. In light of the reviewers' comments, and my own reading further revisions are needed before the paper can be accepted for publication in Plant Direct. Please address all their comment but pay special attention about any reuse of text, figures, or tables. Please let us know if any are meant to be reused, and use citations or rephrasing where appropriate.

Please view the editors' and reviewers' comments below and use their suggestions as a guide while you work on your revision.

When uploading the revised version of this article, please be sure to include the following:

-A word document that contains your response to the reviewers. You should respond to each reviewer comment and note the changes made to the manuscript. If you do not agree with a reviewer's comment and choose not to make a suggested revision, please explain why. Please try to provide as complete an answer as possible to each reviewer's criticisms in the "response to reviewers" section.
-A tracked changes document
- A clean version of the latest version of the manuscript

To upload your revision, please click the link below.
https://plantdirect.msubmit.net/cgi-bin/main.plex?el=A4Lr5Zq2A5nxs2I2A9ftdDE7gBTNFGyOvFeqI3u9YgZ

In order to provide as timely a service as possible, we ask that your revision is resubmitted within three months after receipt of this request. If an extension is needed, please send a request, along with a brief explanation, to the editorial office at plantdirect@wiley.com .


Thank you very much for giving us an opportunity to review your work. I look forward to receiving the next version.


Sincerely,
Larry York
Editor-in-Chief, Plant Direct


---------------------------------------------------------------------------- Reviewer comments:
Reviewer #1:

This manuscript appears to be a continuation of a previous study by same authors which identified SNP/haplotype associations for grain yield and component traits in a sodic and no sodic soil condition. However, there are concerns regarding text recycling and inadequate acknowledgement of sources. The study involves the development and validation of PCR-based markers for the identified SNPs/haplotypes, which holds potential for marker-assisted breeding applications in regular germplasm evaluation.
Some specific comments and observations:
1. The title and abstract need to be modified to reflect what exactly have been done in the current study. What does the 'transferability' refer to?
2. The initial lines of the abstract seem to be borrowed from the previous study by the authors. The abstract should explicitly state that the current study aims to develop and validate PCR-based markers for the previously identified SNPs/haplotypes.
3. It is important to note that genotyping using 8 SNPs may not meet the typical criteria for high throughput genotyping, which usually involves a larger number of markers.
4. The original GWAS study was done using the FIGS population (Sharma et al., 2022), what was the rationale to use this same population for validation?
5. The expression "...gene validation ..." is misleading. In-silicon prediction of gene expression is just a prediction, validation requires cloning or knocking out the gene.
6. "Significance statement
1. The study reports scientific advancement in the progress of marker development for targeted exome-based genotype by sequencing platform (t-GBS) alleles. This is the first report on development of allele-specific molecular markers for SNPs and high linkage disequilibrium haplotypes for the complex trait in wheat." This statement is simply not true.

7. Objective (1): "locate significant de novo SNPs/haplotypes on ~180bp allelic sequence of t-GBS platform with respect to IWGSC RefSeqv1.0" is not supported by the methodology in the current study, it seems this objective has been previously published by the same authors.
8. Figure 2, there were higher than normal number of heterozygous alleles in the allele discrimination plots except for one (PM2: Chr1B). What would be the justification, given those DH and FIGS lines are fixed inbreeds with low degree of heterozygosity?
9. The origin of the Manhattan plot is unclear, as there is no mention of GWAS in the current study. Is the Manhattan plot from a previous analysis? if it is, clearly state it is from previous study; if no, modify the materials and methods part to reflect that.
10. The boxplots also look similar to a previous publication (Sharma et al 2022) except for the swap in the order of the alleles. Why switch the common REF/ALT allele order?
11. Tables 1, 3, and 5 appear to be extracts from a previous publication (Sharma eat al 2022) without proper statement of the source.


Reviewer #2:

Generally, this paper has merit to be published, but it needs further improvement. Specifically, I have some concerns with its flow, structure, and content.
Abstract
• The abstract is lacking some relevant information, including problem statement and research objectives have not been clearly spelt out.
• I would suggest improving or re-structuring the current abstract by starting with justification for conducting the study, followed by clear objective statement (s), brief explanation of the methodology used to achieve the objectives, main findings, and conclusion/recommendation.
• Again in the current abstract version, there is no mention of phenotyping, such as population size tested, experiment design used, and field location where phenotyping was done. Apparently, the authors put more emphasis only on genotyping and marker design than phenotyping, which is one of the most important aspects of the study. When talking about GWAS, you have to cover both phenotyping and genotyping components.
• Besides, I would suggest moving the sentence which starts with, "39 novel SNPs including 18 haplotypes for yield and yield components were identified for sodicity tolerance" down after methodology because this comes under results, so it should come after methodology component.
• More importantly, the authors should consider including the phenotypic variance explained by the most significant SNPs detected via GWAS. Just indicate the range of the phenotypic variance explained.
• A minor correction: Please do not start a sentence with a number, rather write it in words. For example, "Thirty-nine novel SNPs and not "39 novel SNPs" as written in the current version.
Introduction
• For the introduction section, I would suggest including more background information related to the title of the paper. This section should cover all important aspects mentioned in the study.
• Based on previous studies, the authors should elaborate the genetic mechanism of sodic tolerance in wheat or other cereal crops. Sodicity tolerance is one of the main components of this paper and therefore, more detailed background information related to it should be included, such as previous studies done in wheat or other cereal crops related to the present study
• In addition, I would suggest to include more detailed information about allele-specific markers.
• Also, crop name should be accompanied by its scientific name when it is mentioned for the first time. For example, "Bread wheat (Triticum aestivum)
• Please make sure that the abbreviations are written in full throughout the paper when they are mentioned for the first time.
• Genotyping-by-sequencing (GBS) should be explained in detail and the authors also should clearly elaborate the difference between the conventional GBS and t-GBS.
• Overall, the introduction section needs further improvement before publishing.
Materials and Methods
• In the methodology section, more detailed descriptions of the plant materials used in this study should be included.
• Also, I would suggest to include the characteristics of the DH population parents (Scepter and IG107116) and reasons for selecting them to be used in the study.
• Again, FIGS, DH and SNPs should be written in full when mentioned for the first time and this should be corrected throughout the paper.
• The double haploid population was evaluated in one season only, so, I do not think its phenotypic data is robust enough as no genotype-by-environment effect was captured.

Results and discussion
• The chromosome location/number of the most significant SNPs should be indicated in the results section.
• The authors should cite relevant references in the discussion section because some of the sentences do not have their corresponding references.

| **Attachment:** | [Reviewer 1 Review Attachment 1 - 06-29-2023 16:01:28](https://plantdirect.msubmit.net/pd_files/2023/03/04/00001342/00/1_reviewer_attachment_1_1686522536.pdf) |
| --- | --- |

**DECISION LETTER – Round 2**

July 27, 2023
Darshan Sharma
Western Australia Department of Primary Industries and Regional Development Agriculture and Food
3 Baron Hay Court
Bentley Delivery Centre 6151
Australia


MSID: 2023-01146-TWR1
MS TITLE: Exome-based new allele-specific PCR markers and transferability for sodicity tolerance in bread wheat (Triticum aestivum L.)

Dear Dr. Darshan Sharma:

I am pleased to inform you that your manuscript "Exome-based new allele-specific PCR markers and transferability for sodicity tolerance in bread wheat (Triticum aestivum L.)" has been accepted for publication in Plant Direct.

Your article will appear online in the next available issue of Plant Direct. To ensure your article gets published as quickly as possible, please pay attention to the steps detailed below. We have found that most of the delays happen at this stage, especially at the payment stage, so please respond as quickly as possible when prompted.

License Agreement: Once your article has been accepted it will move to Production and undergo admin and file checking - you may receive an email with any queries we have at this stage. When all required items are received by the publisher and queries resolved, the corresponding author will receive an email from Wiley's Author Services system which will ask them to log on at https://authorservices.wiley.com/bauthor and will present them with the appropriate license for completion. Your article cannot be published until both the signed license agreement and payment of the article fee have been received.

Payment of the Open Access Article Publication Fee: All articles published in Plant Direct are fully open access: immediately and freely available to read, download and share. Plant Direct charges a publication fee to cover the publication costs. The corresponding author for this manuscript should have already received a quote from the payments team (cs-openaccess@wiley.com) with the estimated article publication fee; please email cs-openaccess@wiley.com if this has not been received. The corresponding author should log on to the Wiley Author Services site, where the publication fee can be paid by credit card or an invoice. Pro Forma can also be requested. Payment of the publication charge must be received before the article will be published online.

Proofs: You will have the opportunity to look over your paper once more when you receive the author proofs for your article. The proofs will be with you in approximately two weeks. Please note that, in addition to publishing reviewer comments, the author's responses to review comments will also be published alongside the final version of the paper. If you would not like the author's responses to be published, please contact the editorial office at plantdirect@wiley.com .

Promotion of your article: You can help your research get the attention it deserves! Check out Wiley's free Promotion Guide for best-practice recommendations for promoting your work at www.wileyauthors.com/eeo/guide. And learn more about Wiley Editing Services which offers professional video, design, and writing services to create shareable video abstracts, infographics, conference posters, lay summaries, and research news stories for your research at www.wileyauthors.com/eeo/promotion.

Thank you again for your contribution to Plant Direct. If you have any questions, feel free to contact the editorial office at plantdirect@wiley.com .

Sincerely,

Larry York

Larry York

Editor, Plant Direct


---------------------------------------------------------------------------- Editor comments


---------------------------------------------------------------------------- Reviewer comments:

**AUTHORS’ RESPONSE**

**Reviewer 1**

| **No.** | **Comments** | **Response** |
| --- | --- | --- |
| 1. | The title and abstract need to be modified to reflect what exactly have been done in the current study. What does the 'transferability' refer to? | The original title “Exome-based novel SNPs/haplotypes, allele-specific markers, and transferability for sodicity tolerance in wheat” has been changed to “Exome-based new allele-specific PCR markers and transferability for sodicity tolerance in wheat (*Triticum aestivum* L.)”  We claimed transferability of markers because the SNP effect was validated in full FIGS set and one additional DH population (Scepter/IG107116). |
| 2. | The initial lines of the abstract seem to be borrowed from the previous study by the authors. The abstract should explicitly state that the current study aims to develop and validate PCR-based markers for the previously identified SNPs/haplotypes. | Thank you for your comment. The necessary changes in structuring have been made and we hope the abstract now reflects present study. |
| 3. | It is important to note that genotyping using 8 SNPs may not meet the typical criteria for high throughput genotyping, which usually involves a larger number of markers. | High throughput in our study comes from genotyping large number of lines using PCR-based genotyping. |
| 4. | The original GWAS study was done using the FIGS population (Sharma et al., 2022), what was the rationale to use this same population for validation? | The rationale was to check if all the allelic sequences can be amplified. In the present study, not all allelic sequence resulted in successful amplification. We have also validated markers on additional DH population (Line 97-99) |
| 5. | The expression "...gene validation ..." is misleading. In-silicon prediction of gene expression is just a prediction, validation requires cloning or knocking out the gene. | The alleles identified in the study are exome-based. Therefore, validation of allele effect close to the reported genes using SNP marker genotyping and association with phenotype effect served as rapid validation tool. We are aware that more intrinsic validations through molecular techniques are required for confirmation. Considering sodicity as a complex trait with no distinct phenotypes, we relied on present strategies. However, we will carry out comprehensive validation when we receive resources supporting further sodicity research in wheat (Supporting sentences are now added in line 236-241). |
| 6. | Significance statement  1. The study reports scientific advancement in the progress of marker development for targeted exome-based genotype by sequencing platform (t-GBS) alleles. This is the first report on development of allele-specific molecular markers for SNPs and high linkage disequilibrium haplotypes for the complex trait in wheat." This statement is simply not true. | Yes, there are reports on SNP markers developed for alleles of SNP chip, where the SNP location is known and well stated. In the case of t-GBS, new multiple alleles are identified within ~180 bp allele sequence as presented in Supplementary table 1.  We located these SNPs and haplotypes by aligning against ref sequences and the novelty of the work lies here and to our knowledge this is the first report. |
| 7. | Objective (1): "locate significant de novo SNPs/haplotypes on ~180bp allelic sequence of t-GBS platform with respect to IWGSC RefSeqv1.0" is not supported by the methodology in the current study, it seems this objective has been previously published by the same authors. | We have presented methodology for the objective in Line 254- 264. We have also presented supplementary tables showing allelic sequences and precisely mapped allelic locations with respect to reference sequence (IWGSC RefSeqv1.0). |
| 8. | Figure 2, there were higher than normal number of heterozygous alleles in the allele discrimination plots except for one (PM2: Chr1B). What would be the justification, given those DH and FIGS lines are fixed inbreeds with low degree of heterozygosity? | It can be noted that PM2 is a non-homoeologous marker, and all others are homoeologous.  We have added a paragraph in discussion explaining the possible reason for this (Line 189-204) |
| 9. | The origin of the Manhattan plot is unclear, as there is no mention of GWAS in the current study. Is the Manhattan plot from a previous analysis? if it is, clearly state it is from previous study; if no, modify the materials and methods part to reflect that. | The source is now added in figure legend. |
| 10. | The boxplots also look similar to a previous publication (Sharma et al. 2022) except for the swap in the order of the alleles. Why switch the common REF/ALT allele order? | This time only the allelic phenotypic scores of lines with successfully amplified signals were used. The box plots in the present study are presented for all successfully amplified markers, whereas the previous GWAS study had box plots only for alleles with large phenotypic variation.  The favourable allele was represented in first box followed by unfavorable to promote uniform visualizations. |
| 11. | Tables 1, 3, and 5 appear to be extracts from a previous publication (Sharma et al. 2022) without proper statement of the source. | We have added source in titles. The present tables are more refined versions with statistical estimates, details on allelic locations and characterizations and genes closely associated with newly developed SNP markers. |

**Reviewer 2**

| **No.** | **Comments** | **Response** |
| --- | --- | --- |
| 1. | The abstract is lacking some relevant information, including problem statement and research objectives have not been clearly spelt out. | The abstract has now been modified to reflect clarity of present study and outcomes. |
| 2. | I would suggest improving or re-structuring the current abstract by starting with justification for conducting the study, followed by clear objective statement (s), brief explanation of the methodology used to achieve the objectives, main findings, and conclusion/recommendation. | We hope the present changed abstract is convincing. |
| **3.** | In the current abstract version, there is no mention of phenotyping, such as population size tested, experiment design used, and field location where phenotyping was done. Apparently, the authors put more emphasis only on genotyping and marker design than phenotyping, which is one of the most important aspects of the study. When talking about GWAS, you have to cover both phenotyping and genotyping components. | The present study on marker design is derived from the previous GWAS study (Sharma et al. 2022). The present study is about genotyping, and we have phenotyped DH population for validation (details added in Plant materials and DNA extraction under Materials and Methods) |
| **4.** | Besides, I would suggest moving the sentence which starts with, "39 novel SNPs including 18 haplotypes for yield and yield components were identified for sodicity tolerance" down after methodology because this comes under results, so it should come after methodology component. | The abstract is now modified |
| **5.** | More importantly, the authors should consider including the phenotypic variance explained by the most significant SNPs detected via GWAS. Just indicate the range of the phenotypic variance explained. | The phenotypic variance explained by SNPs is now presented in table 3. A statement is also added in abstract (Line 33). |
| **6.** | A minor correction: Please do not start a sentence with a number, rather write it in words. For example, "Thirty-nine novel SNPs and not "39 novel SNPs" as written in the current version. | The change is now made in abstract. |
| **7.** | Introduction For the introduction section, I would suggest including more background information related to the title of the paper. This section should cover all important aspects mentioned in the study. | The focus of this paper was to report SNP marker design for t-GBS data, marker validation, genotyping and variant annotation.  Our previous GWAS publication has all necessary details about sodicity, field screening and other aspects about association mapping. |
| **8.** | Based on previous studies, the authors should elaborate the genetic mechanism of sodic tolerance in wheat or other cereal crops. Sodicity tolerance is one of the main components of this paper and therefore, more detailed background information related to it should be included, such as previous studies done in wheat or other cereal crops related to the present study | The mechanisms of sodicity tolerance have not been exclusively investigated. However, we have discussed general abiotic tolerance mechanisms based on our candidate genes and transcription factors discovery in discussion section. |
| **9.** | In addition, I would suggest to include more detailed information about allele-specific markers. | In introduction, we have a paragraph on allele-specific markers (Line 82-91). |
| **10.** | Also, crop name should be accompanied by its scientific name when it is mentioned for the first time. For example, "Bread wheat (Triticum aestivum) | We have now included scientific name in title  Scientific name is now added in the title of manuscript. |
| **11.** | Please make sure that the abbreviations are written in full throughout the paper when they are mentioned for the first time. | Checked again. Full form of these abbreviations has been mentioned in abstract and introduction |
| **12.** | Genotyping-by-sequencing (GBS) should be explained in detail and the authors also should clearly elaborate the difference between the conventional GBS and t-GBS. | Some modifications/additions to the texts have now been made to reflect the difference between GBS and t-GBS. The difference between GBS and t-GBS are well presented in a paragraph under introduction (Line 71-81) |
| **13.** | Materials and Methods  In the methodology section, more detailed descriptions of the plant materials used in this study should be included. | The plant material and DNA extraction section under Materials and Methods has been updated with necessary details about plant materials used in the study (Line 282-297) |
| **14.** | Also, I would suggest including the characteristics of the DH population parents (Scepter and IG107116) and reasons for selecting them to be used in the study. | The same is now included. |
| **15.** | Again, FIGS, DH and SNPs should be written in full when mentioned for the first time and this should be corrected throughout the paper | Full form of these abbreviations has been mentioned in abstract and introduction |
| **16.** | The double haploid population was evaluated in one season only, so, I do not think its phenotypic data is robust enough as no genotype-by-environment effect was captured. | We have only one year data and these lines were assessed and validated for sodicity performance. |
| **17.** | Results and discussion  The chromosome location/number of the most significant SNPs should be indicated in the results section. | The chromosomal location of significant SNPs has now been highlighted in results (Line 130-131) and further the locations have been mentioned in line 157-167. |
| **18.** | The authors should cite relevant references in the discussion section because some of the sentences do not have their corresponding references. | The necessary references have been added in the manuscript. |
